# 

**Published:** 1973-04

**Authors:** 


Bristol Medico-Chirurgical Journal Vol. 88 (ii) No. 326

### Contents

Edward Jenner — a great Englishman ... ... ... ... ... ... 23

James Macrae

The Medical Library ... ... ... ... ... ... ... ... 29

Bristol Medico-Chirurgical Society ... ... ... ... ... ... ... 30

News and Notes ... ... ... ... ... ... ... ... ... 30

Printed by F. Bailey & Son Ltd., Dursley, Glos.

